# Regulatory Mechanisms and Therapeutic Targeting of PD-L1 Trafficking and Stability in Cancer Immunotherapy

**DOI:** 10.3390/cancers17111747

**Published:** 2025-05-23

**Authors:** Muralidharan Mani, Jeong Woo Park, Thomas F. J. Martin

**Affiliations:** 1 Department of Biochemistry, University of Wisconsin-Madison, Madison, WI 53706, USA; tfmartin@wisc.edu; 2 Basic-Clinic Convergence Research Institute, University of Ulsan, Ulsan 44610, Republic of Korea; jwpark@ulsan.ac.kr

**Keywords:** PD-L1 trafficking, immune checkpoint blockade, immune checkpoint blockade DRG2, TRAPPC4, HIP1R, CMTM6, post-translational modifications, Ras-associated binding proteins (Rab), PD-1 pathway, tumor immune evasion

## Abstract

The PD-L1/PD-1 signaling axis is a pivotal regulator of T-cell activity and a key mechanism by which tumors evade immune surveillance. Inhibiting this pathway has resulted in significant anti-tumor responses, establishing immune checkpoint blockade (ICB) as a crucial component of modern cancer therapy. However, many patients with high PD-L1 expression do not respond to PD-1/PD-L1 blockade, underscoring the necessity for a deeper investigation into the mechanisms underlying this resistance. Recent studies have identified DRG2 as a critical modulator of anti-PD-1 therapeutic efficacy. While DRG2 depletion enhances IFN-γ signaling and increases the overall PD-L1 levels, it disrupts the recycling of endosomal PD-L1, resulting in reduced surface expression and impaired PD-1 interaction, ultimately compromising therapeutic outcomes. Furthermore, TRAPPC4, HIP1R, and CMTM6 help stabilize PD-L1 by preventing lysosome degradation. When depleted, these proteins have been shown to boost the body’s immune response against tumors. Research into the complex regulatory mechanisms of PD-L1 suggests that targeting DRG2, TRAPPC4, HIP1R, and CMTM6 could enhance the effectiveness of PD-1/PD-L1 blockade therapies. This strategy could create exciting new possibilities for cancer immunotherapy and improve patient outcomes.

## Simple Summary

Immune checkpoint therapies that block PD-L1 and its receptor PD-1 have shown promise in treating cancer; however, many patients still do not respond well to these treatments. This lack of response is often due to complex mechanisms that control the level of PD-L1 present on the surface of cancer cells. In this review, we explore how proteins such as DRG2, TRAPPC4, HIP1R, and CMTM6 regulate the movement and stability of PD-L1 within cells. These proteins influence whether PD-L1 is recycled, degraded, or stabilized on the cell surface—factors that directly impact how cancer cells evade the immune system. We also describe how post-translational modifications, such as glycosylation and phosphorylation, affect PD-L1’s behavior. By understanding and potentially targeting these molecular pathways, scientists may improve the effectiveness of cancer immunotherapy and help more patients benefit from treatment.

## 1. Introduction

The programmed death-ligand 1 (PD-L1) and its receptor programmed death-1 (PD-1) are essential components of the immune checkpoint pathway. They play an important role in maintaining immune homeostasis by preventing the over-activation of T cells [1]. However, cancer cells often exploit this pathway to evade immune detection, which can lead to uncontrolled tumor growth [2]. Despite recent advancements in treatment, a significant number of patients, especially those with high PD-L1 expression, do not respond to these therapies. These findings highlight the need for a deeper investigation into resistance pathways [3,4]. Recent research has illuminated the complex regulation of PD-L1 expression and localization, emphasizing the crucial roles of several proteins, including developmentally regulated GTP-binding protein 2 (DRG2), trafficking protein particle complex subunit 4 (TRAPPC4), huntingtin-interacting protein 1-related (HIP1R), and CKLF-like MARVEL transmembrane domain-containing protein 6 (CMTM6) [5,6,7,8]. DRG2 has been established as a crucial regulator of PD-L1 trafficking, affecting its subcellular localization and surface expression. DRG2 depletion increases the overall PD-L1 levels but impairs recycling, resulting in the accumulation of PD-L1 in endosomes and a decrease in surface expression. This disruption weakens the effectiveness of PD-1/PD-L1 therapies [7]. Other research has shown that TRAPPC4 and HIP1R, from the same group, regulate PD-L1 by scaffolding it with RAB11 for recycling and directing it to lysosomes for degradation, respectively. This establishes TRAPPC4 and HIP1R as potential therapeutic targets [5,8]. Moreover, CMTM6 is known to stabilize PD-L1 on the cell surface by preventing its lysosomal degradation, which enhances immune evasion [6]. These findings illustrate the complexity of PD-L1 regulation and suggest that targeting regulatory proteins like DRG2 and CMTM6, in conjunction with immune checkpoint blockade, could effectively combat tumor immune evasion. A deeper understanding of these molecular mechanisms may lead to enhanced therapeutic strategies, potentially helping to overcome resistance to PD-1/PD-L1 inhibitors and improving treatment effectiveness. Therefore, exploring the molecular intricacies that reinforce the necessity for a deeper investigation of PD-L1 regulation shows great promise for advancing cancer immunotherapy and enhancing patient outcomes.

## 2. Therapeutic Modulation of the PD-1 Pathway in Immune Regulation

PD-1 and its ligands, PD-L1 and PD-L2, play a vital role in modulating immune responses. PD-1, discovered in the early 1990s, is an inhibitory receptor found on the surface of T cells [9]. It interacts with its ligands to fine-tune immune responses, ensuring the immune system can attack pathogens while avoiding damage to the body’s tissues. The engagement of PD-1 with its ligands transmits inhibitory signals that suppress T-cell activity, preventing excessive immune responses that could lead to autoimmunity [10]. PD-L1 and PD-L2, the ligands for PD-1, are expressed on various cells, including antigen-presenting cells and some non-immune cells [10]. These interactions are vital in maintaining self-tolerance and preventing autoimmune reactions. For instance, in autoimmune conditions like type 1 diabetes [11] and multiple sclerosis [12], disruptions in the PD-1 pathway can result in a loss of tolerance, allowing self-reactive T cells to attack the body’s own tissues [13].

In chronic infections caused by certain viruses, the expression of PD-1 is increased, which leads to T-cell exhaustion. This exhaustion is marked by a decline in the effector functions of T cells, enabling the pathogen to persist in the host [14,15,16,17]. Research has shown that blocking the interaction between PD-1 and its ligands can rejuvenate these exhausted T cells, restoring their ability to fight the infection effectively [18]. Similarly, many tumors exploit the PD-1 pathway to escape immune surveillance by upregulating PD-L1, which binds to PD-1 on T cells and reduces their ability to attack cancer cells [19]. Therapies that block PD-1 or PD-L1 can reactivate T cells, allowing them to target and destroy cancer cells effectively [20]. Given the crucial role of the PD-1 pathway in regulating immune responses, it presents a promising opportunity for therapeutic intervention. Researchers are exploring various strategies to modulate this pathway for treating autoimmune diseases and chronic infections and to enhance the effectiveness of cancer immunotherapies [21].

In addition to its role in immune suppression, PD-1 signaling plays a critical role in regulating T-cell metabolic programming and determining cellular fate. Upon engagement, PD-1 inhibits the PI3K/Akt/mTOR pathway, shifting cellular metabolism from glycolysis to fatty acid oxidation and oxidative phosphorylation. This promotes the survival of memory-like T cells while limiting terminal exhaustion [22]. Additionally, PD-1 signaling maintains T-cell proliferative potential during chronic antigen exposure by preventing overstimulation-induced apoptosis and limiting the accumulation of terminally differentiated effector cells [23,24].

These mechanisms reveal that PD-1 functions not merely as a negative regulator but as a crucial homeostatic factor that balances effector function with longevity. Post-translational modifications of PD-L1, including glycosylation, phosphorylation, and ubiquitination, predominantly studied in cancer immune evasion contexts, likely influence the PD-1/PD-L1 interaction dynamics and stability at the immunological synapse, thereby modulating the strength and duration of inhibitory signaling [25]. This dual regulatory role suggests that strategic modulation of the PD-1 pathway, rather than complete blockade, might optimize checkpoint inhibitor therapies by reinvigorating effector function while preserving the memory precursor cells that are essential for durable anti-tumor responses.

## 3. Immune Checkpoint Therapy (ICT)

The immune system’s ability to differentiate between healthy and cancerous cells is crucial for effective cancer therapy. The therapeutic modulation of immune checkpoints, such as CTLA-4 and PD-1, as well as their ligands (PD-L1 and PD-L2), has revolutionized cancer treatment by enhancing T-cell responses against tumors. These therapies remove inhibitory signals that prevent T cells from effectively attacking cancer cells [26].

CTLA-4 and PD-1 regulate immune responses through different mechanisms. CTLA-4 primarily limits early T-cell activation by outcompeting CD28 for binding to B7 molecules, while PD-1 inhibits T-cell effector functions within tissues, particularly in the tumor microenvironment [27]. Tumors often exploit these pathways, especially by upregulating PD-L1, which binds to PD-1 on T cells, suppressing their activity and allowing tumors to evade immune destruction [28].

The introduction of immune checkpoint inhibitors, such as anti-CTLA-4 (ipilimumab) [29] and anti-PD-1 (nivolumab, pembrolizumab), has demonstrated significant clinical success in treating various cancers, including melanoma, lung cancer, and renal cell carcinoma [30]. While these inhibitors have dramatically altered the cancer treatment landscape by producing lasting responses and improved survival in certain patients, the inconsistent efficacy across the patient population emphasizes the critical importance of developing biomarkers that can forecast treatment outcomes [3,4]. For instance, PD-L1 expression in tumors has been investigated as a potential biomarker, but its predictive value has been inconsistent across studies [31]. Tumor mutational burden and the presence of neoantigens have also been correlated with response rates, indicating that a tumor’s genetic profile plays a critical role in determining the effectiveness of immune checkpoint blockade [32].

Given the complex nature of immune system interactions with tumors, combination therapies are increasingly viewed as the future of immunotherapy. Combining checkpoint inhibitors with other treatments, such as chemotherapy, radiation, or targeted therapies, may enhance the immune response by creating a more immunogenic tumor microenvironment [33]. Additionally, targeting multiple immune checkpoints simultaneously or combining checkpoint inhibitors with agonists for stimulatory pathways (e.g., ICOS, OX40) can help overcome resistance and achieve more robust anti-tumor responses [34]. The ongoing development of combination therapies and the exploration of additional immune checkpoints and pathways hold promise for extending the benefits of immunotherapy to a broader patient population [34]. When effectively harnessed, the immune system’s adaptability and memory offer the potential for disease control and lasting cures. As research advances, integrating immune profiling, genetic analyses, and novel therapeutic combinations will be crucial in realizing the full potential of immune checkpoint therapies. Furthermore, PD-L1 expression is intricately linked to a network of regulatory mechanisms involving hypoxia-inducible factor 1 alpha (HIF-1α), vascular endothelial growth factor (VEGF), epidermal growth factor receptor (EGFR), and nuclear factor kappa-light-chain-enhancer of activated B cells (NF-κB), all of which play significant roles in cancer progression, immune evasion, and response to therapy. These pathways do not function in isolation but are interconnected, collectively influencing the expression of PD-L1 and, consequently, the ability of tumors to evade immune detection [35].

ICIs have revolutionized cancer therapy, yet they are associated with a wide range of immune-related adverse events (irAEs) that can impact nearly every organ system. Common irAEs include dermatologic reactions such as rash and vitiligo, gastrointestinal toxicities like colitis and diarrhea, endocrine dysfunctions, including hypophysitis and thyroid disorders, and hepatic injury, exemplified by hepatitis [36,37]. Severe but less common complications, such as pneumonitis, myocarditis, and neurological toxicities (including Guillain-Barré syndrome and encephalitis), have the potential to be life-threatening [38,39,40]. The incidence and severity of these irAEs can vary depending on the specific agent used, with combination regimens (e.g., anti-CTLA-4 plus anti-PD-1) showing significantly higher rates of grade ≥ 3 toxicities compared to monotherapy [40]. To mitigate these risks and enhance patient safety, early recognition, risk stratification, and multidisciplinary management are essential.

## 4. Significance of Post-Translational Modifications (PTMs) in PD-L1 Stability and Trafficking

**Figure 1:**
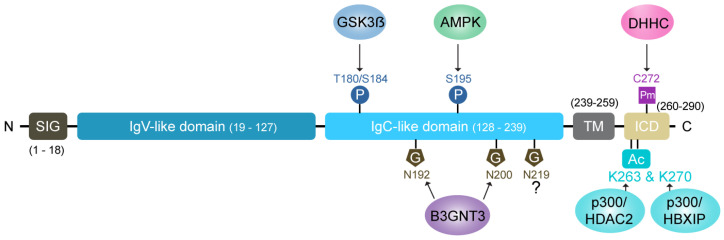
Post-translational modifications (PTMs) regulate PD-L1 trafficking, stability, and function. This schematic illustrates the structural domains of PD-L1 and highlights key post-translational modifications that modulate its intracellular trafficking and stability. PD-L1 consists of a signal peptide (SIG), IgV-like domain (residues 19–127), IgC-like domain (128–239), transmembrane domain (TM), and intracellular domain (ICD). N-linked glycosylation at asparagine residues N192 and N200, mediated by B3GNT3, and at N219 (enzyme unknown, indicated by “?”), stabilizes PD-L1 and enhances its interaction with PD-1. Phosphorylation at threonine and serine residues (T180/S184 by GSK3β and S195 by AMPK) influences PD-L1 degradation and trafficking. Palmitoylation at cysteine residue C272, catalyzed by the DHHC family of palmitoyltransferases, protects PD-L1 from lysosomal degradation and supports its membrane localization. Acetylation of PD-L1 at lysine 263 (K263) by p300 promotes its retention on the cell membrane, while acetylation at lysine 270 (K270) by the HBXIP/p300 complex leads to its accumulation in the cytoplasm. Together, these PTMs finely tune PD-L1’s availability at the cell surface and contribute to immune evasion mechanisms in cancer cells.

The intricate signaling networks that regulate PD-L1 expression underscore the sophisticated relationship between malignancies and immune function. Elucidating these mechanisms at a molecular level is essential for therapeutic innovation. Post-translational modifications (PTMs) emerge within this framework as critical modulators that substantially affect PD-L1 dynamics and transform the tumor microenvironment. These PTMs precisely control PD-L1’s stability, cellular trafficking, and molecular interactions, determining its intracellular processing and extracellular activity—factors that profoundly influence the clinical response to immunotherapies directed at the PD-1/PD-L1 pathway (Figure 1) [35,41,42,43].

### 4.1. Glycosylation: A Key Stabilizing Modification

N-linked glycosylation is one of the most significant PTMs influencing PD-L1 stability. This modification occurs at specific asparagine residues (N192, N200, and N219), extending PD-L1’s half-life from 4 h (non-glycosylated form) to approximately 12 h (glycosylated form) [44]. Enzymes like STT3A actively add core glycans to PD-L1, facilitating its proper folding in the endoplasmic reticulum (ER) [45]. This modification antagonizes glycogen synthase kinase 3 beta (GSK3β)-mediated phosphorylation, which otherwise targets PD-L1 for ubiquitination and proteasomal degradation [44]. Furthermore, glycosylation enhances PD-L1’s binding affinity to its receptor, PD-1, effectively suppressing T-cell activity and enabling immune evasion.

Disruption of glycosylation leads to the retention of PD-L1 in the ER, where it undergoes ER-associated degradation (ERAD). The results demonstrate that glycosylation is critical in preserving PD-L1’s structural stability while enabling proper trafficking through the Golgi apparatus to reach the plasma membrane.

In addition to stability, glycosylation at specific residues is essential for PD-L1’s interaction with PD-1, with inhibitors of N-linked glycosylation impairing this interaction. Targeting the glycosylation pathways represents a promising therapeutic approach to destabilize PD-L1 and enhance immune system-mediated tumor clearance [44,45].

### 4.2. Phosphorylation: A Double-Edged Sword

Phosphorylation dynamically regulates PD-L1’s trafficking and stability by acting as both a stabilizing and destabilizing signal, depending on the context. JAK1-mediated phosphorylation at Y112 facilitates the subsequent glycosylation of PD-L1, promoting its transport from the ER to the Golgi and, ultimately, to the cell surface. This ensures adequate PD-L1 surface expression, which is necessary for its immune-suppressive functions [45].

In contrast, AMP-activated protein kinase (AMPK)-mediated phosphorylation at S195 induces abnormal mannose trimming in the ER, leading to glycosylation defects. This results in PD-L1 retention in the ER and subsequent degradation via ERAD. Similarly, phosphorylation at T180 and S184 by GSK3β targets PD-L1 for ubiquitination and proteasomal degradation. These phosphorylation events are tightly regulated, ensuring that the PD-L1 levels remain balanced under physiological conditions [44].

Phosphorylation’s bidirectional influence establishes it as a critical orchestrator of PD-L1’s cellular journey. Therapeutically, this pathway presents an opportunity for intervention with agents like metformin, which, by activating AMPK, diminishes PD-L1 stability and bolsters anti-tumor immunity through enhanced protein degradation [46].

### 4.3. Ubiquitination and Deubiquitination: Balancing Stability and Degradation

The ubiquitin–proteasome system finely tunes PD-L1 stability through the opposing actions of E3 ligases and deubiquitinating enzymes (DUBs). E3 ligases, such as Cullin-3-SPOP and STUB1, play a key role in ubiquitinating PD-L1, which targets it for proteasomal degradation during the late G1 and S phases of the cell cycle. This degradation is essential for maintaining the homeostasis of PD-L1 and preventing its excessive accumulation, which could result in unregulated immune evasion [47].

**Figure 2:**
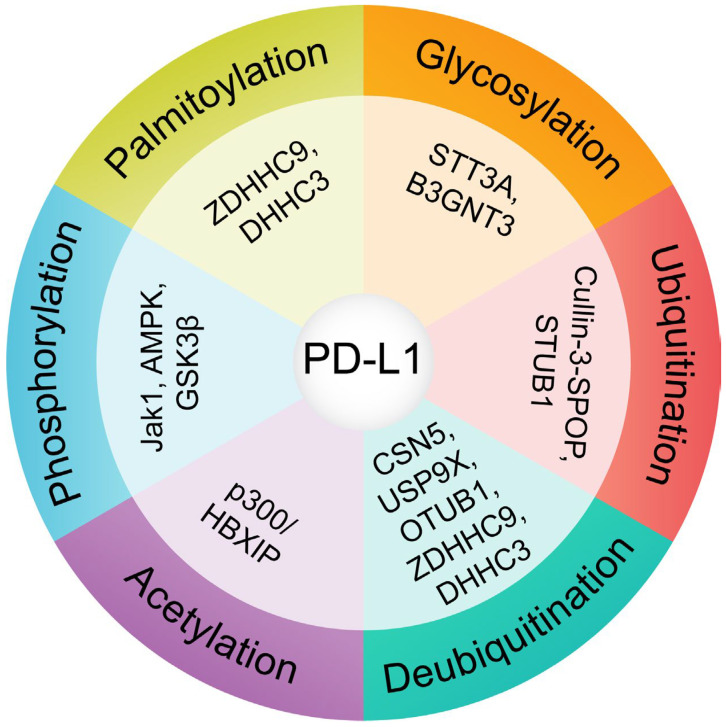
Role of post-translational modifications (PTMs) and their regulatory enzymes in PD-L1 stability. This circular diagram summarizes the major PTMs that modulate PD-L1 turnover and subcellular localization, along with the key enzymes responsible for these modifications. Glycosylation, mediated by STT3A and B3GNT3, stabilizes PD-L1 and enhances its interaction with PD-1. Ubiquitination by E3 ligases such as Cullin-3-SPOP and STUB1 targets PD-L1 for proteasomal degradation. In contrast, deubiquitination by CSN5, USP9X, OTUB1, and others counteracts degradation and prolongs PD-L1 stability. Phosphorylation by kinases, including JAK1, AMPK, and GSK3β, influences both the glycosylation and degradation pathways. Palmitoylation by ZDHHC9 and DHHC3 enhances PD-L1 membrane localization and prevents lysosomal degradation. These PTMs dynamically regulate PD-L1 surface levels and contribute to tumor immune evasion.

Conversely, DUBs such as CSN5 [48,49], USP9X [50], USP22 [51], and OTUB1 [52] counteract this degradation by removing ubiquitin chains from PD-L1, stabilizing the protein (Figure 2). These enzymes are critical in enhancing PD-L1’s half-life and surface expression, particularly in inflammatory cytokine signaling, such as tumor necrosis factor-alpha (TNF-α) [53]. For example, CSN5-mediated deubiquitination has been implicated in cancer cell immune escape by stabilizing PD-L1 during immune responses [48,49].

This balance between ubiquitination and deubiquitination provides a dynamic mechanism to regulate PD-L1 levels. Targeting these pathways, such as inhibiting DUB activity, could serve as a novel strategy to reduce PD-L1 stability and enhance the efficacy of immune checkpoint blockade therapies.

### 4.4. Palmitoylation: Enhancing Surface Retention

Palmitoylation of PD-L1, a lipid-based PTM, is critical for its stability and membrane localization. This modification, mediated by palmitoyltransferases ZDHHC9 and ZDHHC3, occurs at the C272 residue [54]. Palmitoylation prevents PD-L1’s ubiquitination, protecting it from lysosomal degradation and maintaining its surface expression. This modification ensures that PD-L1 remains available to engage PD-1, thereby sustaining immune suppression.

Disruption of palmitoylation through inhibitors such as 2-bromopalmitate (2-BP) reduces PD-L1’s surface levels and sensitizes tumor cells to T-cell-mediated killing. This evidence illuminates palmitoylation’s critical contribution to PD-L1’s functional robustness, identifying it as a promising therapeutic target for disrupting PD-L1 stability and enhancing immune-mediated tumor control [55].

### 4.5. Acetylation of PD-L1 Regulates Subcellular Localization and Accumulation

Protein acetylation is a reversible post-translational modification that regulates protein function by influencing stability, subcellular localization, and protein–protein interactions. In the context of PD-L1, two specific lysine acetylation sites—K263 and K270—have been shown to play distinct roles in immune checkpoint regulation and tumor progression.

Initial evidence indicated that EGF stimulation induces PD-L1 acetylation in A431 cells, suggesting a potential link between growth factor signaling and PD-L1 regulation [56]. Subsequently, acetylation at lysine 263 (K263) by the acetyltransferase p300 disrupts PD-L1’s interaction with HIP1R, a clathrin adaptor that is responsible for its internalization and nuclear translocation [57]. In its unacetylated form, PD-L1 is transported to the nucleus via vimentin and importin-α1, where it acts as a transcriptional co-regulator of the genes involved in MHC class I expression, interferon signaling, and NF-κB activation. Acetylation at K263 prevents this nuclear translocation, retaining PD-L1 at the membrane and ultimately enhancing T-cell-mediated cytotoxicity [57]. This mechanism presents a potential therapeutic strategy to improve the efficacy of PD-1/PD-L1 blockade by limiting PD-L1’s immunosuppressive nuclear functions.

In parallel, regulatory mechanisms HBXIP (hepatitis B X-interacting protein) promotes PD-L1 acetylation at lysine 270 (K270) by recruiting p300. Acetylation at K270 leads to the cytoplasmic accumulation of PD-L1 and increases its overall protein levels in breast cancer cells [58]. Clinically, HBXIP expression positively correlates with PD-L1 levels, and HBXIP knockdown significantly reduces tumor growth, underscoring the therapeutic relevance of the HBXIP–p300–PD-L1 axis. This pathway may serve as a biomarker-guided therapeutic target in tumors exhibiting high expression of both HBXIP and PD-L1.

Although both K263 and K270 are acetylated by p300, it remains unclear whether HBXIP influences acetylation at K263—an open question that warrants further investigation. Collectively, these findings provide new insights into the regulation of PD-L1 beyond immune checkpoint interaction and suggest that targeting PD-L1 acetylation could serve as a strategy to enhance the efficacy of checkpoint blockade or overcome resistance. Notably, the site-specific and functional diversity of PD-L1 acetylation distinguishes it from other modifications, such as glycosylation or ubiquitination. Continued exploration of this pathway may lead to novel therapeutic combinations and improved immunotherapy outcomes.

## 5. Integration and Therapeutic Implications

The interplay between various PTMs—glycosylation, phosphorylation, ubiquitination, and palmitoylation—determines PD-L1’s intracellular trafficking, stability, and surface localization. These modifications collectively orchestrate PD-L1’s lifecycle, ensuring its availability for immune evasion while maintaining homeostasis within the tumor microenvironment.

Therapeutic interventions targeting PTMs have shown promise in modulating PD-L1 stability and function. For instance, metformin-mediated activation of AMPK or palmitoylation inhibitors can reduce PD-L1 levels, enhancing the immune clearance of tumor cells. Disrupting the glycosylation machinery with inhibitors like 2-deoxyglucose impairs PD-L1’s interaction with PD-1, sensitizing tumors to immune checkpoint blockade [59]. Future research should aim to unravel the molecular “trafficking code” of PD-L1, including the roles of SNAREs and small GTPases, to identify new therapeutic opportunities.

By targeting PTMs, it may be possible to fine-tune PD-L1’s stability and trafficking (Table 1), overcome resistance to PD-1/PD-L1 therapies, and improve cancer patients’ outcomes.

## 6. Intracellular Trafficking and Cell Surface Expression of PD-L1

**Figure 3:**
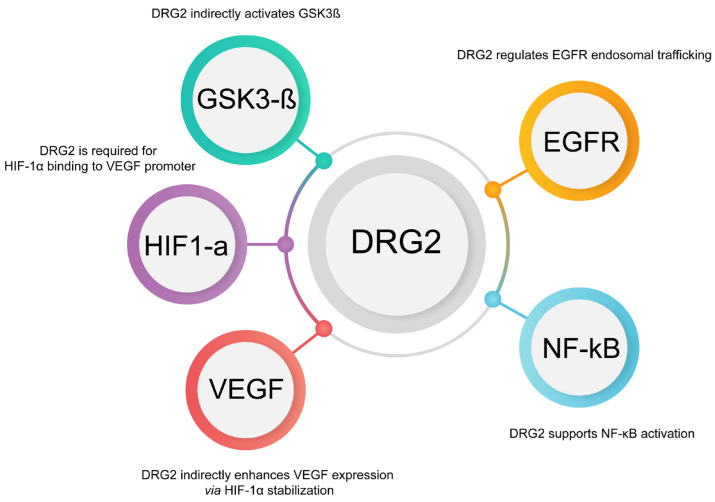
DRG2 regulates key signaling pathways involved in PD-L1 stability and expression. This circular schematic summarizes the role of developmentally regulated GTP-binding protein 2 (DRG2) in modulating upstream regulators that influence PD-L1 trafficking, degradation, and surface expression. DRG2 affects the EGFR and VEGF pathways, both of which modulate PD-L1 expression and trafficking. It also regulates hypoxia-inducible factor 1α (HIF-1α), which contributes to PD-L1 stabilization under hypoxic conditions. DRG2-mediated suppression of GSK3β prevents the phosphorylation-dependent degradation of PD-L1, enhancing its stability. Additionally, DRG2 modulates NF-κB signaling, which promotes PD-L1 transcription and prevents its degradation. These interactions position DRG2 as a central node in controlling PD-L1 availability and immune evasion in tumors.

While PTMs exert significant control over PD-L1 stability, the dynamic localization of PD-L1 at the cell surface is critically dependent on its intracellular trafficking. PD-L1, upon ligand binding, undergoes receptor-mediated endocytosis. Subsequently, it resides within early endosomes. Endosomal maturation, facilitated by the Rab5-to-Rab7 GTPase transition, directs PD-L1 towards either lysosomal degradation or recycling back to the plasma membrane via a Rab11-dependent pathway. This complex process involves a coordinated interplay of various proteins, including those involved in vesicle formation, transport, and fusion. Proteins such as CMTM6, which stabilizes PD-L1, HIP1R, which may influence PD-L1 endocytosis, and components of the TRAPP complex and DRG2, essential for intracellular vesicle transport, collectively contribute to the intricate trafficking and, ultimately, the cell surface expression of PD-L1 (Figure 3).

### 6.1. DRG2’s Role in Membrane Trafficking and PD-L1 Dynamics

Various cellular pathways tightly regulate the trafficking and expression of PD-L1, an important immune checkpoint protein. PD-L1 plays a crucial role in tumor immune evasion, making it a key target in cancer therapy. Understanding how its expression and localization are controlled can lead to more effective therapeutic strategies. One such regulator is DRG2, which, while not directly involved in PD-L1 expression, influences several pathways that modulate the PD-L1 levels and trafficking, including the EGFR [61], NF-κB [62], HIF-1α, and VEGF pathways [63].

DRG2 is a GTP-binding protein that plays a critical role in stabilizing membrane tubules. These tubules are essential for the trafficking of various receptors, including the transferrin receptor (TfR) [64]. By stabilizing these membrane structures, DRG2 ensures the proper recycling of receptors to the plasma membrane, which is crucial for maintaining their availability and function [65].

**Figure 4:**
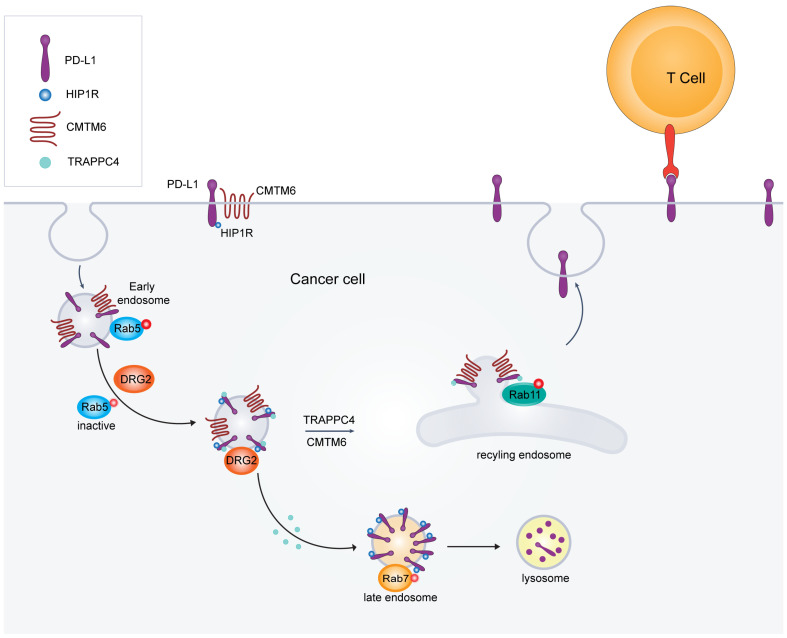
Intracellular trafficking and regulation of PD-L1 by DRG2, TRAPPC4, HIP1R, and CMTM6 in cancer cells. This schematic illustrates the endocytic trafficking route of PD-L1 and the roles of regulatory proteins in its intracellular localization and stability. Upon internalization from the plasma membrane, PD-L1 enters early endosomes marked by Rab5. DRG2 facilitates the stabilization of endosomal tubules, promoting PD-L1 sorting and trafficking. PD-L1 may be directed toward late endosomes marked by Rab7 for lysosomal degradation or recycled back to the plasma membrane via Rab11-positive recycling endosomes. TRAPPC4 and CMTM6 promote the recycling of PD-L1 to the cell surface, supporting immune evasion by maintaining PD-L1 availability for interaction with PD-1 on T cells. In contrast, HIP1R directs PD-L1 to lysosomes for degradation. CMTM6 also stabilizes PD-L1 on the plasma membrane by preventing its degradation. Together, these pathways tightly regulate PD-L1 localization and surface expression, which influence the tumor’s ability to suppress T-cell-mediated immune responses.

Given its role in receptor trafficking, DRG2 may similarly influence the trafficking of PD-L1 (see Figure 4). For PD-L1 to interact effectively with PD-1 on T cells, it must be properly localized to the cell surface, allowing tumor cells to evade immune responses. If DRG2 stabilizes the membrane tubules involved in PD-L1 trafficking, then its depletion or dysfunction could lead to altered PD-L1 localization. This could result in a reduced presence of PD-L1 on the cell surface, potentially impacting the tumor’s ability to evade the immune system [7].

#### 6.1.1. Indirect Regulation of PD-L1 Expression by DRG2 Through EGFR and NF-κB

EGFR is a well-known regulator of PD-L1 expression in various cancers, particularly in non-small cell lung cancer (NSCLC). When EGFR is activated, it triggers downstream signaling pathways, including the PI3K/AKT and NF-κB pathways, which can enhance the transcription of PD-L1 [66]. DRG2 may play a role in this process by modulating the trafficking and recycling of EGFR, ensuring its proper localization and function at the cell membrane. If DRG2 is essential for maintaining the stability and recycling of EGFR-containing vesicles [61], its activity could directly influence EGFR signaling and, consequently, PD-L1 expression.

NF-κB is a transcription factor that is activated by various signals, including those from EGFR [66]. It directly regulates the expression of PD-L1 by binding to its promoter to increase transcription, thereby contributing to tumors’ evasive immune properties. By influencing the trafficking of EGFR, DRG2 indirectly modulates NF-κB activity and PD-L1 expression.

#### 6.1.2. Interaction with HIF-1α and VEGF Pathways

HIF-1α is a transcription factor that becomes stabilized under hypoxic conditions, which are commonly found in the tumor microenvironment. It is known to upregulate VEGF (vascular endothelial growth factor), promoting the formation of new blood vessels (angiogenesis), and directly increasing PD-L1 expression by binding to hypoxia response elements (HREs) within the PD-L1 promoter [67].

In this context, DRG2 may play a critical role by stabilizing membrane structures that are essential for the trafficking of receptors and signaling molecules involved in the HIF-1α pathway. For example, VEGF, upregulated by HIF-1α, is a central player in angiogenesis and also modulates immune responses within the tumor microenvironment. DRG2’s involvement in receptor trafficking may extend to the modulation of VEGF receptor (VEGFR) trafficking, thereby influencing VEGF signaling and impacting HIF-1α activity. By stabilizing receptor-containing vesicles, DRG2 could help ensure that signaling pathways, such as those mediated by HIF-1α and VEGF, are effectively maintained, which may indirectly affect PD-L1 expression.

Furthermore, DRG2 influences PD-L1 expression and function through its role in stabilizing membrane tubules and facilitating the proper trafficking of critical receptors, including EGFR (epidermal growth factor receptor) [61], TfR (transferrin receptor) [64], and possibly PD-L1 itself [7]. By modulating the trafficking of EGFR, DRG2 affects downstream pathways, such as NF-κB, which directly regulate PD-L1 transcription. Additionally, DRG2’s impact on the HIF-1α/VEGF axis introduces another level of regulation, linking hypoxia, angiogenesis, and immune evasion (Figure 3).

This complex interplay emphasizes DRG2’s critical importance in cancer pathophysiology, functioning not just in receptor transport but comprehensively in tumor immune evasion processes. Therapeutic approaches directed at DRG2 or its regulatory networks may offer promising strategies to amplify PD-L1 inhibitor effectiveness and improve clinical responses. A deeper investigation into how DRG2 governs these pathways will be fundamental for creating targeted interventions that undermine tumors’ immune evasion capabilities.

### 6.2. HIP1R-Mediated Lysosomal Targeting of PD-L1

Wang et al., investigates the regulatory mechanisms by which HIP1R influences the degradation of PD-L1 within lysosomes and its subsequent effects on T-cell-mediated cytotoxicity. The study establishes HIP1R as a key negative regulator of PD-L1, mediating its effect through direct binding that targets PD-L1 for lysosomal degradation. Specifically, HIP1R interacts with PD-L1 through its conserved C-terminal region and directs it to lysosomes via an intrinsic sorting motif. The investigators identified HIP1R as a fundamental element in regulating PD-L1 turnover through comprehensive genomic and proteomic methodologies [5]. The study further revealed that a di-leucine sorting signal can be utilized to create a lysosomal targeting molecule for delivering a transmembrane protein, such as PD-L1, to lysosomal degradation. The lysosomal degradation involving the HIP1R di-leucine signal necessitates Alix and the endosomal sorting complex required for transport (ESCRT) complex, indicating that it is a ubiquitin-independent process. Typically, the internalization of cargo proteins to the multi-vesicular body (MVB) or lysosome depends on ESCRT complexes. The subunits of the ESCRT-0 and ESCRT-I complexes possess ubiquitin-binding domains, allowing them to transfer ubiquitinated cargo proteins to ESCRT-III for internalization [68]. Alternatively, cargo proteins can also bind to the Alix protein in a manner that does not involve ubiquitination, facilitating their interaction with ESCRT-III [69]. The HIP1R di-leucine signal represents a significant ubiquitin-independent pathway that shows the ESCRT machinery’s functional flexibility. While protein ubiquitination often serves as the primary tag for lysosomal degradation, the ESCRT complex can also recognize and facilitate cargo protein degradation through other sorting signals, particularly di-leucine motifs (see Figure 4). The investigation demonstrates that HIP1R inactivation in multiple cancer cell lineages promotes PD-L1 accumulation, augmenting its cell surface expression and attenuating T-cell-mediated tumor cytotoxicity effectiveness. Conversely, overexpressing HIP1R results in decreased levels of PD-L1, indicating that HIP1R could be a potential therapeutic target for modulating immune evasion in cancer [5].

Moreover, the study identifies specific sequences within HIP1R that are essential for interacting with PD-L1 and its role in lysosomal sorting. The researchers developed a chimeric peptide called PD-LYSO, based on the “binding-sorting” model derived from HIP1R’s mechanisms. This peptide effectively targets PD-L1 to lysosomes, promoting its degradation in cancer cells [5].

The study suggests that manipulating HIP1R activity through direct targeting or utilizing engineered peptides like PD-LYSO could offer a new approach to enhancing the effectiveness of immune checkpoint blockade therapies by reducing the PD-L1 levels and overcoming immune resistance [5].

Both HIP1R and DRG2 act as upstream modulators of PD-L1 localization and function. They interact not only with PD-L1 but also with a broader range of endocytic and signaling machinery. HIP1R, a clathrin-adaptor protein, plays a key role in the internalization and lysosomal degradation of PD-L1 by connecting it to the actin cytoskeleton and endocytic vesicles. This process is enhanced by phosphorylation at Ser929, a Pin1-dependent event that increases HIP1R’s affinity for actin. When HIP1R is lost, PD-L1 accumulates on the cell surface, leading to impaired immune clearance in models of colon cancer [5].

In addition, DRG2 influences several upstream signaling pathways, including EGFR, NF-κB, and HIF-1α/VEGF, and also regulates Rab5 inactivation, which is crucial for endosomal trafficking and PD-L1 recycling. By functioning at the intersection of transcriptional regulation and intracellular transport, both HIP1R and DRG2 serve as complex regulators of PD-L1 expression and immune evasion. Their dual roles make them promising therapeutic targets for enhancing the efficacy of checkpoint inhibitors.

### 6.3. TRAPPC4: A Critical Regulator of PD-L1 Trafficking and Tumor Immune Evasion

This study examines the complex regulation of PD-L1 trafficking and stabilization, explicitly focusing on the roles of TRAPPC4, HIP1R, and PTMs. These mechanisms collectively contribute to tumor immune evasion. By keeping PD-L1 on the surface of tumor cells, these regulatory pathways facilitate immune suppression, allowing tumor cells to avoid destruction by cytotoxic T cells. This comprehensive analysis sheds light on PD-L1 biology and identifies potential therapeutic strategies to enhance anti-tumor immunity.

TRAPPC4, a core subunit of the trafficking protein particle (TRAPP) complex, emerges as a crucial regulator of PD-L1 recycling [8]. As a molecular scaffold, TRAPPC4 facilitates the interaction between PD-L1 and RAB11 within recycling endosomes. This interaction ensures that internalized PD-L1 is efficiently recycled back to the tumor cell surface instead of being directed toward lysosomal degradation (see Figure 4). Maintaining PD-L1 on the plasma membrane is essential for interacting with PD-1 on T cells, which is vital for immune evasion, as it suppresses T-cell activation and cytotoxicity [70]. Experimental evidence from loss-of-function screening and mass spectrometry has identified TRAPPC4 as a significant regulator of PD-L1 expression. Depleting TRAPPC4 results in a notable reduction of PD-L1 levels, both in vitro and in vivo, as demonstrated in murine models. This depletion leads to increased infiltration of CD8+ T cells into tumor sites and enhances T-cell-mediated cytotoxicity [8].

On the other hand, overexpressing TRAPPC4 improves PD-L1 recycling, making tumor cells more resistant to immune attacks and more responsive to PD-L1 blockade therapies. These findings establish TRAPPC4 as a key regulator of immune evasion and a promising therapeutic target. Targeting TRAPPC4 may attenuate PD-L1 recycling and synergize with immune checkpoint blockade to enhance anti-tumor immunity [8].

In contrast to TRAPPC4’s stabilizing role, HIP1R acts as a negative regulator of PD-L1 by promoting its lysosomal degradation. HIP1R interacts with PD-L1 through specific lysosomal targeting sequences, facilitating its transport to lysosomes for degradation. This process lowers PD-L1 levels on the tumor cell surface, reducing its immunosuppressive effects and enhancing T-cell-mediated immune responses. Depleting HIP1R leads to the accumulation of PD-L1 at the plasma membrane, further suppressing T-cell activity and enabling tumor cells to evade immune destruction. Conversely, enhancing HIP1R activity or mimicking its function presents a promising therapeutic strategy. For example, synthetic peptides like PD-LYSO, designed to replicate HIP1R’s lysosomal targeting role, have shown potential in promoting PD-L1 degradation and enhancing the effectiveness of immune checkpoint inhibitors [5].

The regulation of PD-L1 is influenced by PTMs, including glycosylation, ubiquitination, phosphorylation, and palmitoylation.

Each modification plays a unique role in modulating PD-L1 stability, trafficking, and function. N-linked glycosylation, for instance, stabilizes PD-L1 by preventing its degradation through the proteasome and enhancing its interaction with PD-1. This modification extends PD-L1’s half-life and amplifies its immunosuppressive effects [71]. However, abnormal glycosylation can trap PD-L1 in the ER, leading to its degradation through the ERAD pathway. Ubiquitination is another regulatory mechanism where E3 ligases such as Cullin3 and β-TrCP tag PD-L1 for degradation [44]. This process is counterbalanced by deubiquitinases like CSN5, which stabilize PD-L1 by removing ubiquitin chains [49]. Phosphorylation further influences PD-L1 trafficking and often intersects with the glycosylation and ubiquitination pathways. For example, AMPK-mediated phosphorylation can modulate glycosylation patterns, affecting PD-L1 transport from the ER to the Golgi and subsequently to the plasma membrane [46]. Moreover, palmitoylation prevents PD-L1 internalization and degradation, ensuring its prolonged presence on the plasma membrane [42].

TRAPPC4, HIP1R, and PTMs orchestrate the dynamic regulation of PD-L1, enabling tumor cells to fine-tune their expression and sustain immune suppression. The functional crosstalk among these pathways facilitates immune escape and pinpoints precise molecular vulnerabilities for therapeutic exploitation. By inhibiting TRAPPC4 to disrupt PD-L1 recycling, enhancing HIP1R-mediated lysosomal degradation, or targeting post-translational modifications to destabilize PD-L1, we can explore promising avenues to improve the efficacy of immune checkpoint therapies. This integrative understanding of PD-L1 regulation provides a strong framework for developing next-generation cancer immunotherapies to overcome resistance mechanisms and achieve lasting anti-tumor responses.

### 6.4. CMTM6 and CMTM4: Key Regulators of PD-L1 Stability and Their Implications for Enhancing Cancer Immunotherapy

The regulation and stabilization of PD-L1, a critical immune checkpoint protein, are essential for tumor immune evasion. PD-L1 suppresses T-cell activation by binding to PD-1, which inhibits anti-tumor immune responses and promotes tumor progression. Burr et al., identifies CMTM6 and its homolog CMTM4 as vital regulators of PD-L1 stability and surface expression, providing important insights into the mechanisms that tumors exploit to evade immune destruction [6].

CMTM6, in particular, emerges as a principal regulator of PD-L1, preventing its degradation through the ubiquitin–proteasome pathway. It binds directly to PD-L1 at the plasma membrane and within recycling endosomes, shielding it from ubiquitination mediated by STUB1, an E3 ubiquitin ligase [72]. This protective mechanism ensures that PD-L1 is recycled back to the plasma membrane rather than directed toward the lysosomal or proteasomal degradation pathways. By maintaining PD-L1 levels on the cell surface, CMTM6 facilitates its continuous interaction with PD-1, effectively suppressing T-cell activity [6]. Studies have demonstrated that the loss of CMTM6 results in a dramatic reduction in PD-L1 expression, significantly impairing the tumor’s ability to evade immune responses. This stabilization is crucial for sustaining an immunosuppressive environment and supporting tumor growth [73].

CMTM4, closely related to CMTM6, plays a secondary role by acting as a compensatory regulator when CMTM6 is absent. Though less effective than CMTM6, CMTM4 helps preserve PD-L1 stability, contributing to a robust regulatory system that ensures the persistence of PD-L1 on tumor cells. The concurrent presence of these proteins demonstrates their cooperative function in promoting immune evasion, reflecting the multifaceted and redundant architecture of this regulatory network [74].

The regulation of PD-L1 by CMTM6 and CMTM4 is post-translational; neither protein influences PD-L1 transcription or its trafficking from the ER to the cell surface (see Figure 4). Their actions aim to stabilize PD-L1 at the plasma membrane and protect it from endosome degradation. These proteins stabilize PD-L1 at the plasma membrane and prevent its endosomal degradation. Notably, CMTM6 exhibits exclusive specificity for PD-L1, unaffected by other immune regulators like MHC class I or PD-L2, making it a promising and precise therapeutic target. Moreover, CMTM6 also regulates PD-L1 in tumor-infiltrating immune cells, such as macrophages and dendritic cells, further amplifying its role in creating an immunosuppressive tumor microenvironment [72].

This study employed advanced methodologies, including genetic screens using CRISPR-Cas9, to identify and validate the roles of CMTM6 and CMTM4 in PD-L1 regulation [74]. These findings provide a strong basis for targeting these proteins in cancer therapy. Disrupting CMTM6 function, for example, could destabilize PD-L1, weakening tumor immune evasion and enhancing the T-cell-mediated destruction of cancer cells. Such an approach could complement existing PD-L1/PD-1 blockade therapies, overcoming resistance mechanisms and improving treatment outcomes [74].

The intricate regulatory roles of DRG2, TRAPPC4, HIP1R, and CMTM6 in PD-L1 trafficking and stability present compelling opportunities for therapeutic intervention to enhance anti-tumor immunity. Inhibition of CMTM6 may accelerate PD-L1 lysosomal degradation, potentially sensitizing immunologically “cold” tumors to checkpoint blockade, as demonstrated in multiple solid tumor models. HIP1R-mediated endocytosis reduces PD-L1 surface expression, and its functional loss leads to immune checkpoint resistance in pancreatic and triple-negative breast cancer models, highlighting its therapeutic relevance [75]. Targeting TRAPPC4 may disrupt the vesicular recycling machinery essential for PD-L1 re-expression at the plasma membrane. Furthermore, selective inhibition of DRG2 function could impair PD-L1 recycling and Rab5-dependent endosomal transport, thereby reducing PD-L1 presentation at the immunological synapse. Strategically combining these trafficking-directed therapies with conventional PD-1/PD-L1 inhibitors may provide synergistic benefits by blocking ligand–receptor interactions and limiting total PD-L1 availability, potentially overcoming adaptive resistance and enhancing durable responses across diverse cancer types.

## 7. Conclusions and Future Directions for Targeting PD-L1 Trafficking

PD-L1 plays a central role in tumor immune evasion, primarily through its interaction with PD-1 on T cells. This interaction suppresses the immune response and allows tumors to grow unchecked. Several essential proteins, including DRG2, TRAPPC4, HIP1R, and CMTM6, regulate the trafficking and stability of PD-L1. These proteins contribute to PD-L1’s ability to remain on the tumor cell surface and evade immune detection (Figure 4).

DRG2 has been identified as a critical regulator of PD-L1 trafficking. It influences the recycling process, ensuring that PD-L1 is appropriately localized to the cell surface. Disruption of DRG2 leads to the mislocalization of PD-L1, reducing its surface presence and, consequently, the efficacy of PD-1/PD-L1 blockade therapies. Targeting DRG2 could enhance the effectiveness of these therapies by ensuring that PD-L1 is correctly trafficked and exposed to the tumor cell surface, where it can be effectively targeted.

TRAPPC4 and HIP1R, identified in other studies, also play significant roles in PD-L1 regulation. TRAPPC4 functions as a scaffold for PD-L1 and RAB11, coordinating the recycling of PD-L1 and protecting it from lysosomal degradation. HIP1R, conversely, directs PD-L1 to lysosomes for degradation, thus reducing its surface expression. Targeting these proteins could provide novel strategies for reducing the PD-L1 levels on the tumor surface, thereby enhancing T-cell-mediated cytotoxicity and improving the efficacy of immune checkpoint inhibitors.

CMTM6 preserves PD-L1 expression on the cell surface by shielding it from lysosomal degradation. Targeting CMTM6 could reduce PD-L1 abundance in tumor cells, diminishing their capacity for immune evasion.

The strategic inhibition of multiple factors—DRG2, TRAPPC4, HIP1R, and CMTM6—represents a compelling approach to regulate PD-L1 surface levels on cancer cells, potentially reversing immune resistance mechanisms and amplifying the effectiveness of PD-1/PD-L1 blockade strategies. Subsequent investigations should prioritize developing selective inhibitors that target these proteins and evaluate their therapeutic potential alongside established immune checkpoint inhibitors to achieve more potent and sustained anti-tumor effects in immunity.

A complex network of signaling pathways, post-translational modifications, and intracellular trafficking machinery governs PD-L1 expression and localization regulation. While therapies targeting the PD-1/PD-L1 axis have revolutionized cancer immunotherapy, challenges such as variable response rates and immune-related toxicities remain. Targeting PD-L1 regulators like DRG2, TRAPPC4, HIP1R, and CMTM6 presents an opportunity to fine-tune PD-L1 availability and improve the therapeutic response. Because multiple factors influence the stability and trafficking of PD-L1, further identification of these regulatory components may enable more precise therapeutic strategies that enhance efficacy while minimizing immune-related adverse effects.

## Figures and Tables

**Table 1:** Summary of post-translational modifications regulating PD-L1 stability and trafficking.

Post-Translational Modification (PTM)	Regulator/Enzyme	Effect on PD-L1	Therapeutic Implications
Glycosylation	STT3A [45]	Enhances PD-L1 stability and proper folding. Protects against degradation by preventing GSK3β binding.	Disruption of glycosylation destabilizes PD-L1 and reduces its immune-suppressive function.
	B3GNT3 [60]	Facilitates PD-L1 interaction with PD-1.	N-linked glycosylation inhibitors impair PD-L1/PD-1 binding, enhancing anti-tumor immunity.
Phosphorylation	JAK1 [4,45]	Facilitates glycosylation and trafficking to the cell surface.	Essential for maintaining PD-L1 surface expression.
	AMPK [46]	Promotes abnormal glycosylation and ER retention, leading to degradation.	AMPK activators (e.g., metformin) promote PD-L1 degradation and enhance T-cell-mediated killing.
	GSK3β [44]	Targets PD-L1 for degradation via phosphorylation.	Potential target to modulate PD-L1 levels in tumor cells.
Ubiquitination	Cullin-3-SPOP [47]	Marks PD-L1 for proteasomal degradation.	Enhancing this pathway can reduce PD-L1 stability and improve immunotherapy efficacy.
	STUB1 [47]	Promotes PD-L1 ubiquitination and degradation.	Prevents PD-L1 accumulation in tumor cells.
Deubiquitination	CSN5 [49], USP9X [50], OTUB1 [52]	Stabilizes PD-L1 by removing ubiquitin chains.	Inhibition of DUBs can reduce PD-L1 levels and enhance immune checkpoint blockade therapies.
Palmitoylation	ZDHHC9, ZDHHC3 [54,55]	Maintains PD-L1 stability and surface expression. Prevents lysosomal degradation.	Inhibitors like 2-bromopalmitate destabilize PD-L1, sensitizing tumor cells to immune responses.
Acetylation	p300 [57]	Decrease the stability	Blocks nuclear translocation. Increase membrane retention
	HBXIP/p300	Increase the stability	Cytoplasmic accumulation.
